# Optimizing the Malaxation Conditions to Produce an Arbequina EVOO with High Content of Bioactive Compounds

**DOI:** 10.3390/antiox10111819

**Published:** 2021-11-17

**Authors:** Alexandra Olmo-Cunillera, Julián Lozano-Castellón, Maria Pérez, Eleftherios Miliarakis, Anna Tresserra-Rimbau, Antònia Ninot, Agustí Romero-Aroca, Rosa Maria Lamuela-Raventós, Anna Vallverdú-Queralt

**Affiliations:** 1 Department of Nutrition, Food Science and Gastronomy, XIA, Faculty of Pharmacy and Food Sciences, Institute of Nutrition and Food Safety (INSA-UB), University of Barcelona, 08028 Barcelona, Spain; alexandra.olmo@ub.edu (A.O.-C.); julian.lozano@ub.edu (J.L.-C.); mariaperez@ub.edu (M.P.); leytmil@gmail.com (E.M.); annatresserra@ub.edu (A.T.-R.); lamuela@ub.edu (R.M.L.-R.); 2 CIBER Physiopathology of Obesity and Nutrition (CIBEROBN), Institute of Health Carlos III, 28029 Madrid, Spain; 3 Laboratory of Organic Chemistry, Faculty of Pharmacy and Food Sciences, University of Barcelona, 08028 Barcelona, Spain; 4 Institute of Agrifood Research and Technology (IRTA), Fruit Science Program, Olive Growing and Oil Technology Research Team, 43120 Constantí, Spain; Antonia.Ninot@irta.cat (A.N.); Agusti.Romero@irta.cat (A.R.-A.)

**Keywords:** polyphenols, carotenes, olive oil quality, ripening index, Mediterranean pattern, multivariate statistics

## Abstract

To meet the growing demand for high-quality extra-virgin olive oil (EVOO) with health-promoting properties and pleasant sensory properties, studies are needed to establish optimal production parameters. Bioactive components of EVOO, including phenolic compounds, carotenoids, chlorophylls, tocopherols, and squalene, contribute to its organoleptic properties and beneficial health effects. The aim of this study was to develop an Arbequina EVOO with high phenol content, particularly oleocanthal and oleacein, on a laboratory scale by analyzing the effects of different temperatures (20, 25, and 30 °C) and times (30 and 45 min) of malaxation. Higher temperatures decreased the levels of the phenolic compounds, secoiridoids, tocopherols, and squalene, but increased the pigments. EVOO with the highest quality was produced using malaxation parameters of 20 °C and 30 min, although oleocanthal and oleacein were higher at 30 and 25 °C, respectively. Overall, 20 °C and 30 min were the processing conditions that most favored the physiological and chemical processes that contribute to higher levels of bioactive compounds in the oil and diminished their degradation and oxidation processes.

## 1. Introduction

The organoleptic and health-promoting properties that define high-quality EVOO are associated with a high content of phenolic and volatile compounds, which can be affected by the production process. The mechanical extraction of olive oil involves crushing the olives into a paste, which then undergoes malaxation, a mixing process in which small oil droplets progressively coalesce, facilitating the separation of the oil from the aqueous phase [1]. To promote coalescence and therefore obtain higher oil yields, the viscosity of the paste can be reduced by increasing the malaxation temperature, although the oil quality can suffer if it is excessively high [2]. On average, the malaxation process takes 45 to 60 min, depending on the characteristics of the olive, and may be increased to maximize the oil extraction. However, higher times can be offset by a reduction in some nutritional properties of EVOO if the atmosphere in the headspace of the mixer contains oxygen [1]. Thus, malaxation of the olive paste may be viewed not only as an extraction process to achieve satisfactory yields but also as a crucial production step whose modification may enhance both the quality and quantitative chemical composition of the EVOO [1]. The influence of malaxation time [3,4,5] and temperature [5,6,7,8] on the overall quality of EVOO has been widely investigated but with divergent results.

The oxidative stability and flavor of EVOO, as well as its nutritional properties and health effects, are associated with a high content of fatty acids (97–98% of the total weight of EVOO), many of them monounsaturated, mainly oleic acid, and other valuable minor components [9]. Among the minor components, phenolic compounds are responsible for the health effects attributed to EVOO, as demonstrated in epidemiological studies in which the consumption of EVOO enriched with polyphenols was correlated with a cardioprotective effect in Mediterranean populations [10]. Besides their antioxidant activity, phenolic compounds are responsible for the pungency and bitterness of EVOO [11]. Among this family of bioactive compounds, secoiridoids are the major group, being oleacein and oleocanthal the most abundant and highly desired due to their organoleptic and health-promoting properties [12]. Therefore, obtaining an EVOO with high content of these two secoiridoids is of great interest. Carotenoids and chlorophylls give the oil a yellow-green color and contribute to its oxidative stability [11].

As malaxation temperature and duration are crucial parameters in the production of EVOO of optimum quality and antioxidant potential, the aim of this study was to investigate the interactive effects of these factors on the content of phenolic compounds, pigments, tocopherols, and squalene in the oil, giving special attention to the secoiridoids oleocanthal and oleacein. Olive oils were produced from “Arbequina” olives, a traditional cultivar native to Catalonia, Spain, using two different malaxation times (30 and 45 min) and three temperatures (20, 25, and 30 °C), and differences in their chemical composition were examined.

The ripening index (RI) also influences the content of the bioactive compounds [13]. Numerous studies have demonstrated that the phenolic content of the olive fruit decreases during ripening [3,14], as does the chlorophyll level [7]. The olive samples used in this experiment had different RIs. Since the aim of this study was to evaluate the effect of the temperature and time of malaxation, the effect of the RI was eliminated with the statistical analyses.

## 2. Materials and Methods

### 2.1. Reagents

Cyclohexane and 0.1 N sodium thiosulfate (Na_2_S_2_O_3_) was purchased from Carlo Erba Reagents (Val-de-Reuil, France); acetic acid, chloroform, methanol (MeOH), and acetonitrile (ACN) from Sigma-Aldrich (Madrid, Spain); potassium iodide (KI) from Honeywell Fluka (Buchs, Switzerland); hexane, sodium hydroxide pellets (NaOH), starch 1% and phenolphthalein from Panreac (Castellar del Vallès, Spain), and ethanol 96% from VWR Chemicals (Fontenay-sous-Bois, France). Ultrapure water was obtained using a Milli-Q purification system (Millipore, Bedford, MA, USA).

Regarding the standards, oleocanthal (≥95% purity) was purchased from Merck (Darmstadt, Germany); oleacein and oleuropein aglycone (≥90% and 95% purity, respectively) from Toronto Research Chemical Inc. (ON, Canada). Luteolin (≥96% purity), oleuropein (98% purity), pinoresinol (≥95% purity), squalene, and (±)-α-tocopherol (≥96% purity) were acquired from Sigma-Aldrich. Apigenin and *p*-coumaric (>98% purity) were from Fluka, and hydroxytyrosol was from Extrasynthese (Genay, France).

### 2.2. Olive Oil Production

The olive oils were produced in the second week of November 2019 from olives of the Arbequina cultivar grown in Catalonia. The olives were collected from trees with greener fruits, and the oil was produced on the day of harvest. Since olives came from different olive trees, the RI was calculated for a sample of olives coming from each olive tree, following the methodology described in Uceda and Frías [15]. The orchard is in the Institute of Agrifood Research and Technology (IRTA) in Constantí (Tarragona), which is sited at latitude 41.172° N and longitude 1.169° E with 100 m altitude and 15 km from the Tarraconense coast. The climate is the typical Mediterranean with high environmental humidity (60–70%), average annual temperature of 15.8 °C, and 500 mm rainfall and that occurs mainly in April–May and September. The soil is narrow (40–50 cm) and has a loamy texture, a basic pH (8.1), and a 4% content of active limestone, with little fertility. Cultural practices at the orchard are usual in the producing area, and irrigation is supplied.

Six different olive oils were produced in an ABENCOR system, varying the malaxation time (30 and 45 min) and temperature (20, 25, and 30 °C). Three replicates were produced for all conditions. First, olives were washed with water, and leaves and branches were removed. The olives were then crushed using a sieve of 5 mm, and the resulting olive paste was malaxed in the ABENCOR system, controlling the temperature of the water and the time. After malaxation, the olive paste was centrifuged to separate the oil from the solid and water phases. Finally, the oil phase was decanted to remove residual solid particles, which were centrifuged again to recover any remaining oil. The obtained oil was collected, filtered with a filter paper, and stored at −20 °C until analyzed.

### 2.3. Determination of Olive Oil Quality Parameters

K_232_, K_270,_ and ∆K were determined following the methodology described in the Commission Regulation (EEC) No. 2568/91 [16].

The peroxide value was determined as follows. A total of 30 mL of a solution of acetic acid and chloroform (3:2) and 0.5 mL of saturated KI were added to 5 g of olive oil. After mixing, 30 mL of water was added. The titration was performed with 0.1 M Na_2_S_2_O_3_ until the olive oil solution turned yellow. Immediately, 0.5 mL of starch 1% was added, and the solution was titrated until the blue/purple color vanished [17].

The acidity was determined as follows. A total of 45 mL of ethanol was added to 7.05 g of olive oil, followed by 50 µL of phenolphthalein. This solution was titrated with 0.025 M NaOH until the color changed slightly to light pink [17].

### 2.4. Extraction and Determination of the Phenolic Fraction

The isolation of the phenolic fraction was performed by liquid-liquid extraction. A total of 0.5 g of olive oil was dissolved in 1 mL of hexane in a 10 mL centrifuge tube and shaken for 30 s. A total of 2 mL of MeOH:H_2_O (8:2) was added, and the samples were shaken again for 30 s. Afterwards, the two phases were separated by centrifuging the samples at 3000 rpm and 4 °C for 4 min. The methanolic fraction was collected in another centrifuge tube and underwent a second cleaning with 1 mL of hexane, whereas the hexane fraction was again treated with 2 mL of MeOH:H_2_O (8:2) to recover the remaining phenolic compounds. All tubes were shaken for 30 s and centrifuged at 3000 rpm and 4 °C for 4 min. The methanolic phases were recovered and concentrated under reduced pressure. Finally, the phenolic extracts were reconstituted with 800 µL of ACN and stored at −80 °C until analyzed.

The identification and quantification of individual phenolic compounds were carried out by liquid chromatography coupled to mass spectrometry in tandem mode (LC-MS/MS) following the methodology described in López-Yerena et al. [18] and Lozano-Castellón et al. [19] with few modifications. An Acquity TM UPLC (Waters; Milford, MA, USA) coupled to an API 3000 triple-quadrupole mass spectrometer (PE Sciex, Concord, ON, Canada) with a turbo ion spray source was used. The column and precolumn were an Acquity UPLC^®^ BEH C18 column (2.1 × 50 mm, i.d., 1.7 µm particle size) and Acquity UPLC^®^ BEH C18 Pre-Column (2.1 × 5 mm, i.d., 1.7 µm particle size) (Waters Corporation^®^, Wexford, Ireland), respectively. Two methods were used: method (a) for the identification of oleacein, oleocanthal, and ligstroside and oleuropein aglycone [19], and (b) for the identification of other phenolic compounds [18].

For method (a), the mobile phases used were MeOH (A) and H_2_O (B), both with 0.1% formic acid. An increasing linear gradient (*v*/*v*) of A was used (t (min), %A), as follows: (0, 5); (2, 5); (4, 100); (5, 100); (5.50, 5); (6.5, 5). For method (b), the mobile phases were ACN (A) and H_2_O with 0.05% acetic acid (B). An increasing linear gradient (*v*/*v*) of A was used (t (min), %A), as follows: (0, 2); (2, 5); (7.5, 40); (7.6, 100); (8.5, 100); (8.6, 5); (9, 2), (10, 2). Both methods had a constant flow rate of 0.6 mL/min, an injection volume of 5 µL, and the temperature of the column was 50 °C.

Ionization, in negative mode, was performed using electrospray ionization (ESI), and all the compounds were monitored in the multiple monitoring mode (MRM) with the settings described in López-Yerena et al. [18] and Lozano-Castellón et al. [19]. The system was controlled by Analyst version 1.4.2 software supplied by ABSciex, and the chromatograms were integrated using the same software.

### 2.5. Determination of Pigments, Tocopherols, and Squalene

Pigments (chlorophylls and carotenoids) were determined by spectrophotometry, following the methodology described in Minguez-Mosquera et al. [20] with some modifications. A total of 1.5 g of olive oil was weighted in a 5 mL volumetric flask and made up to the mark with cyclohexane. Before measuring, the samples were filtered with a 0.2 µm filter. Absorbance was measured at 670 and 470 nm for chlorophylls and carotenoids, respectively, using a UV-3600, UV-VIS-NIR spectrophotometer (Shimadzu Corporation, Japan).

200 µL of the same sample dilution was diluted in 800 µL of cyclohexane for the determination of tocopherols and squalene by liquid chromatography, an Acquity UPLC coupled to a photodiode array detector (PDA) (Waters Corporation^®^, Milford, MA, USA). The column was an Atlantis^®^ T3 (2.1 × 100 mm, i.d., 3 µm particle size) (Waters Corporation^®^, Wexford, Ireland). The mobile phases used were ACN (A) and MeOH (B) in an increasing linear gradient (*v*/*v*) of B as follows: (t (min), %B): (0, 30); (15, 30); (17, 100); (40, 100); (41, 30); (47, 30), at a constant flow rate of 0.4 mL/min. The injection volume was 10 µL, and the column temperature was 40 °C. The PDA measured the absorbance at 295 nm and 210 nm for total tocopherols and squalene, respectively.

### 2.6. Statistical Analysis

All malaxation treatments were produced by triplicate, as well as the determination of the quality parameters and bioactive compounds.

A principal component analysis (PCA) was performed using SIMCA 13.0.3 to assess the impact of the different variables on our EVOO samples and see how they were distributed. The PCA indicated that the RI had a considerable impact on the phenolic profile of the olive oil, confirming previous reports [7,13,21]. The values of the RI were different depending on the olive tree from which the olives were collected (Table A1). The values ranged from 1.16 to 2.26. Therefore, in order to eliminate the effect of this variable and focus only on the temperature and time of malaxation, results were adjusted taking into account the RI of the olive samples, which was performed in three groups (1.16–1.20, 1.44–1.54, and 2.20–2.26), as we have previously classified [13]. Afterwards, the statistical analyses to see the effect of temperature and time of malaxation were performed. Statistical analyses were conducted using STATA software (version 16.0; StataCorp, College Station, TX, USA), and the test was a nonparametric kernel regression.

## 3. Results and Discussion

### 3.1. Determination of Olive Oil Quality Parameters

The quality parameters of olive oil can indicate changes in quality induced by the production processes. The primary oxidation of polyunsaturated fatty acids (PUFA) results in conjugated hydroperoxides and diene-conjugated products, which can be measured with the peroxide value and the extinction coefficient of K_232_, respectively. The secondary oxidation gives triene conjugated systems that can be measured with the extinction coefficient of K_270_ [22]. ∆K correlates with the state of oxidation [23]. The acidity is used to determine the deterioration of the oils due to the hydrolysis of triacylglycerides [22].

Independently of the malaxation conditions, all the oils met the quality parameters required for EVOO status (Table 1) according to Commission Regulation (EEC) No. 2568/91 [16] (acidity ≤ 0.8 g oleic acid/100 g, peroxide value ≤ 20 mEq O_2_/kg, K_232_ ≤ 2.50, K_270_ ≤ 0.22, ∆K ≤ 0.01).

The acidity increased slightly with temperature and was not affected by time. K_270_ and peroxide values were not significantly affected by temperature (*p* > 0.05) although K_270_ tended to decrease with time (*p* = 0.049), and peroxide values to increase (*p* = 0.048). In contrast, K_232_ increased slightly with higher temperatures and time. The temperature had a similar effect on ∆K, but no differences were detected over malaxation time.

Other studies reported an increase in acidity, peroxide values, and K values only at high temperatures (>30 °C) due to an increase in the lipase activity and oxidation processes [2,7,24], whereas no significant changes were detected at lower temperatures [25]. Nevertheless, other studies did not find a clear effect of the temperature on these parameters [4].

Although malaxation time (<75 min) is reported not to affect acidity, peroxide values, or K values [4,26], malaxing for 90 min resulted in olive oils with higher acidity [14] due to lipolytic activity. Additionally, the effects of these parameters can vary according to the cultivar [4]. Kalua et al. [5] concluded that peroxide and K values were not discriminating variables for malaxation conditions. In the current study, no great differences were found, although the slight increase in the acidity with higher temperatures suggests an enhanced lipase activity. Malaxation at the range of temperatures and times studied did not increase oxidation processes. However, the peroxide value and K_232_ showed a tendency to increase over time, suggesting that the longer malaxation processes triggered the primary oxidation of PUFA. In summary, even though the quality parameters did not differ markedly under the conditions studied, the results indicate that EVOOs produced by malaxation with a lower temperature and duration (20 °C and 30 min) have fewer imperfections.

### 3.2. Determination of the Phenolic Fraction

Table 2 shows the concentration of the phenolic compounds identified in the EVOO samples. Table 3 shows the statistical data once the results were adjusted for the RI. As mentioned before, results were adjusted for the RI in order to eliminate the effect of this variable and only focus on the effect of the variables temperature and time of malaxation. From now on, all the results discussed in this section will refer to the adjusted data (Table 3). When the results were adjusted for the RI, the β values showed a clear tendency for all the phenolic groups (Table 3). Negative β values indicate a decreasing tendency, whereas positive β values indicate an increasing tendency.

#### 3.2.1. Total Polyphenols

Significant differences (*p* < 0.05) in total polyphenol concentrations were observed for all the tested malaxation conditions (Table 3). The levels had a clearly decreasing tendency with higher temperatures and longer times, being the β values −7.95 and −18.19 when comparing 25 to 20 °C and 30 to 20 °C, respectively, and −8.10 when malaxation was extended from 30 to 45 min. This behavior can be expected as the degradation of polyphenols is accelerated by heat and exposure to oxygen [9,27].

In the literature, longer malaxation is mainly reported to reduce phenolic content [3,4,26,28], and only a few studies describe a limited effect [2,5,21]. The decrease in polyphenols during malaxation can be explained by two phenomena: (a) the activity of oxidoreductases (polyphenol oxidase (PPO) and peroxidase (POD)), and hydrolytic enzymes (*β*-glucosidase) [9,29], and (b) the transfer of phenols to the water phase, as they have a more hydrophilic character [30]. When using a sealed malaxer, Polari et al. [29] reported an increase in phenolic content with longer malaxation (75 min), which led to the subsequent oxygen depletion inhibiting oxidoreductase activity. At that point, a longer process may increase the phenolic transfer from water to oil. Furthermore, the effect of malaxation time also seems to depend on the ripeness of the fruit, having a greater impact if the olives are in an early maturation stage when they have a higher phenolic content [3]. Therefore, the different ripening indices of the olive samples could explain the high or low effect of the malaxation time in different publications.

The effect of the malaxation temperature is more controversial, some studies finding the correlation with phenolic content to be negative [7,24], as in our case, and others positive [2,3,4,31]. Different factors could be responsible for this discrepancy: agro-climatic conditions, the olive cultivar, the RI of the fruit, the experimental scale (industrial or laboratory), and the temperature and time ranges of malaxation [24].

As well as phenolic content, olive fruits of different varieties and RI vary in enzymatic activity [32] and will, therefore, react differently to the conditions of malaxation [21]. Results will also differ if the malaxation is performed in a laboratory rather than an oil mill plant [2,4], as the smaller quantities of olive paste allow more contact with atmospheric oxygen, facilitating oxidation of the phenolic compounds.

The phenolic content of the final product also depends on the equilibrium between different processes during malaxation. These can be positive phenomena, which enhance phenolic content in the oil, such as the release of phenolic compounds from the cellular tissues and their solubility in the oil phase, or negative, such as degradation by chemical or enzymatic oxidation [32]. The temperature and time of malaxation play a complex role in this balance. According to Parenti et al. [25], the temperature both promotes phenolic degradation and improves their solubility in the oil. By increasing the temperature, the viscosity of the olive paste is reduced and facilitates polyphenol transfer from the solid to the liquid phase, and also the partition coefficient is increased, which allows more phenols to move from the water to the oil phase [2,30]. It has also been suggested that higher temperatures enhance the release of polyphenols from the fruit tissues [27,33]. On the other hand, increasing the temperature usually promotes the degradative activity of oxidoreductases and *β*-glucosidases [1,9], as does oxygen or the exposure of olive paste to air [29]. Nevertheless, the optimum temperature for these enzymes and their thermal stability varies among cultivars [27]. Taticchi et al. [27] concluded that the oil produced from cultivars with the most thermally stable enzymes had the lowest increase in phenols.

Accordingly, the progressive reduction in phenolic content in the current study could be because oxidoreductases were more active at 25 and 30 °C than at 20 °C, and a longer malaxation (45 versus 30 min) provided them with more oxygen and more time to develop their activity. Furthermore, longer processing could favor the transfer of the phenolic compounds from the oil to the water phase. To confirm these hypotheses, further research on the enzymatic activity and phenolic content of the water phase is required.

#### 3.2.2. Secoiridoids

As the major group of phenolic compounds in olive oil, secoiridoids were expected to show similar behavior to that of total polyphenols: the higher the temperature and the longer the malaxation, the less secoiridoids would remain in the oil. Indeed, β values of the total secoiridoids were −8.00 and −18.25 when the temperature was increased to 25 and 30 °C, respectively, and −8.15 when malaxation was extended from 30 to 45 min (Table 3). Studies show that secoiridoids are the phenolic group most affected by oxidative degradation [27] and temperature [6]. A depletion of secoiridoids with malaxation time and temperature has been attributed to the action of oxidoreductases [4,14,33].

In comparison with 20 °C, the respective β values of ligstroside aglycone, oleuropein aglycone, and elenolic acid were −10.59, 4.63, and −1.56 after malaxation at 25 °C, and −21.13, −9.35, and −3.16 at 30 °C (Table 3). Conversely, oleocanthal was positively affected by the temperature increase, its β value being 8.65 and 16.6 when applying 25 and 30 °C, respectively, whereas oleacein was not significantly affected (*p* < 0.05). Longer malaxation had a negative impact on ligstroside aglycone, oleuropein aglycone, oleacein, and elenolic acid, whose β values were −3.58, −2.43, −5.75, and −1.09, respectively, when the process was extended from 30 min to 45 min, while oleocanthal was again positively affected (β = 4.75). Similar results have been reported by Gómez-Rico et al. [33].

Although variable results can be found in the literature, most studies agree that *o*-diphenols or secoiridoid derivatives of hydroxytyrosol are more affected by oxidase activity than those derived from tyrosol, which is attributed to the substrate specificity of PPO and possibly also of POD [34]. In accordance with our results, secoiridoid derivatives from hydroxytyrosol, including oleacein and oleuropein aglycone, have been negatively correlated with malaxation temperature and time in laboratory-scale conditions [14,27,33]. Another study in an oil mill plant found that at 30 °C, *o*-diphenols decreased over time [26]. Lukić et al. [21] reported higher levels of oleocanthal and oleacein and lower levels of oleuropein aglycone and ligstroside aglycone at 30 °C than at 21 °C. Diamantakos et al. [3] also found that oleocanthal and oleacein increased with temperature.

However, as mentioned earlier, the effect of malaxation conditions may vary depending on the RI of the olives. Lukić et al. [21] reported that oleocanthal and oleacein increased when malaxing olives of medium RI at 21 °C for 60 versus 30 min, yet both secoiridoids diminished when the RI was high. Additionally, Diamantakos et al. [3] suggested that temperatures of 30 °C could enhance the activity of biosynthetic enzymes for the formation of oleocanthal and oleacein. Yet oxidoreductases also show optimal activity at around 30 °C, so secoiridoid oxidation will increase at this temperature. Therefore, the reduction in secoiridoids observed in our study could have been caused by oxidative reactions catalyzed by PPO, POD, and lipoxygenase, which are promoted by longer exposure of olive paste to air.

Once again demonstrating the influence of the cultivar, Boselli et al. [31] reported that a higher temperature increased the content of oleuropein and ligstroside aglycone in EVOO produced from a mix of Frantoio and Leccino olives but had a negative effect with Coratina.

A study in an oil mill plant using Arbequina olives from Catalonia [28] also found that oleacein levels decreased with malaxation time, whereas the more stable structures of ligstroside derivatives were less affected by enzymatic activities. A low degradation rate could, therefore, also be responsible for the increase in oleocanthal in our study, favoring its transfer to the oil phase. In contrast, Gómez-Rico et al. [33] did not find any clear trend for oleocanthal when using Cornicabra olives. In Arbequina olive oil, secoiridoid transfer to the oil phase increased with the RI, despite a lower concentration in the fruit [28]. In agreement with our results, Kalua et al. [5] concluded that a short processing time (30 min) favors the formation of oleacein over its degradation, whereas higher temperatures (30, 45, and 60 °C) combined with longer times (60, 90, and 120 min) promotes the degradation rate.

Although the biosynthesis of secoiridoids in the olive fruit has not been fully elucidated, it is known that oleacein and oleocanthal are formed during oil production by the action of *β*-glucosidase and esterases and that oleuropein aglycone and ligstroside aglycone may act as their respective precursors [35], a pathway supported by our results. The decrease in oleuropein aglycone and ligstroside aglycone with temperature and time indicates they were degraded and/or transformed to oleacein and oleocanthal, respectively. Oleacein was not significantly affected by temperature but decreased with time, unlike oleocanthal, probably because oxidoreductases have more affinity for oleacein. The apparent non-effect of a higher temperature on oleacein levels suggests that its biosynthesis and oxidation might occur at a similar rate, considering that the enzymes involved in both processes share an optimal temperature of around 30 °C. On the other hand, the decrease in oleacein when the malaxation time was extended could be explained by longer exposure to air, which would accelerate the oxidoreductive degradation.

Conversely, the increase in oleocanthal with temperature and time could indicate its biosynthesis occurred at a greater rate than its degradation due to the lower affinity of oxidoreductases for this compound. The formation of oleocanthal was favored by the increased activity of the biosynthetic enzymes at 30 °C and a longer time for the reaction to take place. Moreover, the higher depletion of ligstroside aglycone could suggest it was transformed into oleocanthal to a greater extent than oleuropein aglycone into oleacein. Finally, the concentration of elenolic acid, which may have been expected to increase, being a secoiridoid degradation product, decreased with higher temperatures and longer malaxation, albeit less so than other secoiridoids. These conditions may have favored the transfer of this compound to the water phase as it has a more hydrophilic character.

Thus, the major secoiridoids, oleacein, and oleocanthal, differed in their behavior during the malaxation process. The EVOO with the highest oleacein content was obtained with 30 min of malaxation, regardless of the temperature (20, 25, or 30 °C), whereas 30 °C and 45 min produced the EVOO with the highest oleocanthal content. Considering the total secoiridoids, the highest content could be obtained by malaxation at 20 °C for 30 min.

#### 3.2.3. Minor Compounds: Flavones, Phenolic Acids, Phenolic Alcohols, and Lignans

Although no significant differences were found when the results were adjusted for the RI (*p* > 0.05), phenolic acids and phenolic alcohols both showed a tendency to decrease with temperature (negative β values), whereas flavones and lignans tended to increase (positive β values). Only phenolic alcohols decreased with a longer malaxation time, while flavones, lignans, and phenolic acids were enhanced (Table 3).

Results in the literature are contradictory. Marx, Casal et al. [6] observed an increase in flavones with temperature, but no trend for phenolic alcohols and acids, whereas other studies found no changes in the flavone content [14,33]. Boselli et al. [31] reported that phenolic acids and alcohols were not significantly affected by malaxation temperature and time. An increase in phenolic alcohols with temperature and time has been attributed to the hydrolysis of secoiridoids [7,25,36] and was more visible at temperature ranges above those tested here (>30 °C). Likewise, an increase in flavones could be attributed to the transformation of its glycosylated forms into the aglycones, increasing its solubility in the oil [13]. Contrary, phenolic acids showed a negative correlation with higher temperatures (20−60 °C) [36].

Jiménez et al. [14] also found that hydroxytyrosol and tyrosol decreased with longer malaxation, which favors oxidoreductase activity as well as the diffusion of phenols into the aqueous phase, and observed little effect on phenolic acids. A decrease in hydroxytyrosol and tyrosol was similarly reported by Lukić et al. [21]. In our EVOO samples, tyrosol was not detected, whereas hydroxytyrosol was not significantly affected by temperature and increased only slightly when the malaxation was extended to 45 min. This small increment in hydroxytyrosol could be related to the oxidation of its derivative oleacein, which decreased slightly at 45 min, but was not affected by temperature, as described above.

Among the phenolic compounds, lignans are the most lipophilic [28] and have the lowest antioxidant activity [25]. In our and other studies [14,21,25], the absence of any significant changes in lignans, which tended to increase with time and temperature, is likely due to their scarce oxidation and easy solubility in the oil phase.

### 3.3. Pigments, Tocopherols, and Squalene

Pigments (chlorophylls and carotenoids) are responsible for the color of olive oil and contribute to its oxidative stability [37]. The concentrations of carotenes and chlorophylls in our EVOO samples (Table 4) increased with temperature, which promotes the release of pigments from plant tissues [4,8,25,33]. However, studies carried out at above 30 °C did not observe any increase due to the intensification of their degradation [8]. The pigment content is also reported to increase with time, having longer to transfer to the oily phase [26], although they are also susceptible to degradation by lipoxygenase. The final content in the oil, therefore, depends on the balance between transfer and degradation. In our study, we observed that at 20 and 25 °C, the pigment concentration increased with time, suggesting a higher rate of release and transfer than degradation. However, at 30 °C, the content decreased with time, indicating that longer exposure to a higher temperature may accelerate degradation.

Tocopherols, also known as vitamin E, coexist in four different forms (α-, β-, γ-, and δ-) in plant-based foods, all acting as antioxidants. The predominant form in olive oil is α-tocopherol [38]. Measuring all the tocopherols together, we found a decrease when the temperature was increased from 20 to 25 °C, and no significant differences between 25 and 30 °C, whereas the increment of time from 30 to 45 min had a positive effect on their concentration. In contrast, an increase in temperature has been reported [2,8], attributed to a higher release from the fruit tissues [8]. Inarejos-García et al. [2] did not find any significant changes, although values increased slightly with temperature. Regarding time, Jiménez et al. [14] found a slight increment at 90 min compared to 45 min. Tocopherols are strong antioxidants that protect PUFA from oxidative damage [38], so an oxidation process during malaxation could have caused their depletion in our study.

Squalene is the main lipophilic hydrocarbon of olive oil and has been linked to some of its beneficial effects [39]. We observed a diminishing content with temperature, as did Seçmeler and Üstündağ [40], but an increase with time. Its unsaturated molecule makes squalene unstable and easily oxidized [39]. In addition, it protects PUFA against temperature-dependent autoxidation [41]. Both factors could explain the depletion at higher temperatures. The quality parameters measured in our study related to oxidation did not change, which suggests PUFA may have been protected from oxidation by the action of squalene, together with tocopherols and phenolic compounds. Squalene is found in a free form in the lipid bilayer [40], so longer malaxation may promote its release from the cells to the oil.

### 3.4. Principal Component Analysis (PCA)

A PCA was performed with all the data collected (quality parameters, phenolic compounds, pigments, tocopherols, and squalene) to assess how the EVOO samples were distributed and which variable or variables caused it.

**Figure 1:**
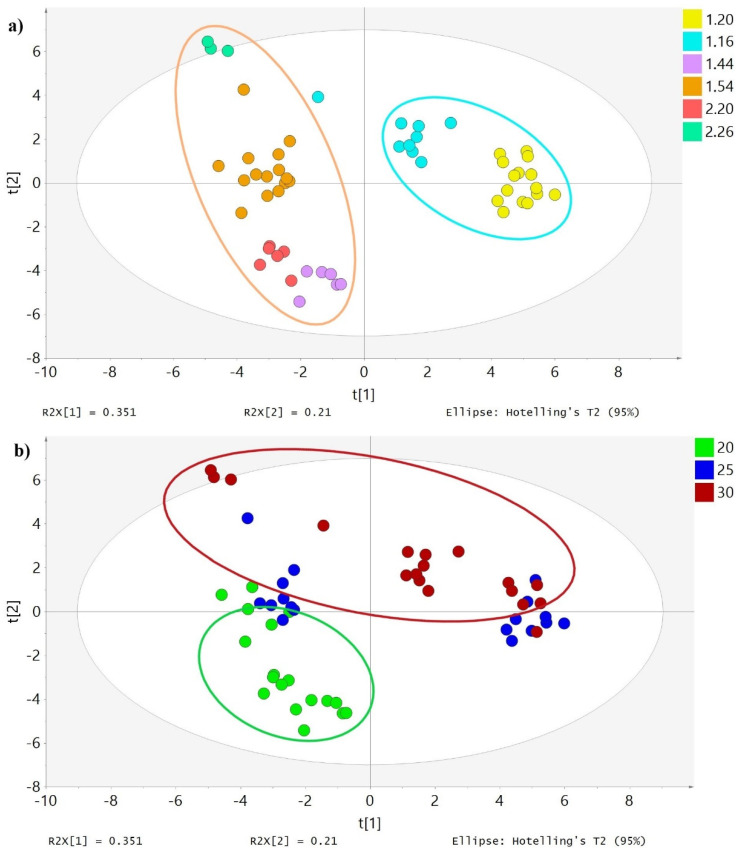
Score scatter plot of the EVOO samples. Figure (**a**) shows the EVOO samples colored according to their RI (1.20, 1.16, 1.44, 1.54, 2.20, and 2.26). Figure (**b**) shows the EVOO samples colored according to the malaxation temperature (20, 25, and 30 °C).

In the PCA, the distribution of olive oil samples reveals the considerable impact of the RI (Figure 1a). In fact, their distribution on the X axis (principal component) is mainly influenced by the RI, the factor that separates the samples. Those with the lowest RI are located to the right of the X axis (1.20 and 1.16), whereas those with higher values are on the left. Analysis of the second principal component shows that the next most influential variable is temperature (Figure 1b). Oils malaxed at 20 °C are located below the Y axis, while those malaxed at 25 and 30 °C are mainly above. The time of malaxation did not affect the distribution of the samples when the two principal components were analyzed, indicating the impact of this variable was low compared to the RI and malaxation temperature or insignificant.

**Figure 2:**
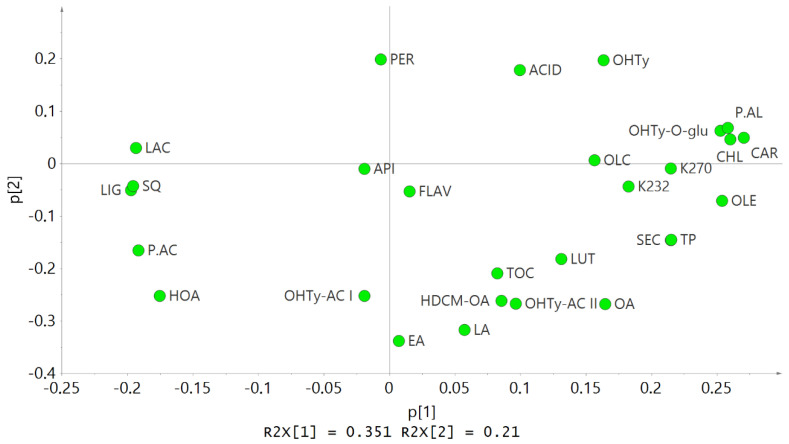
Loading scatter plot showing the distribution of the different parameters analyzed in the EVOO samples. PER: Peroxide value; ACID: Acidity; CAR: Carotenes; CHL: Chlorophylls; TOC: Tocopherols; SQ: Squalene; TP: Total phenols; SEC: Secoiridoids; LIG: Lignans; FLAV: Flavones; P.AL: Phenolic alcohols; P.AC: Phenolic acids; API: Apigenin; LUT: Luteolin; LAC: Lactone; EA: Elenolic acid; OHTy: Hydroxytyrosol; OHTy-AC I, -AC II: Hydroxytyrosol acetate; OHTy-O-glu: Hydroxytyrosol-O-glucoside; HOA: Hydroxyoleuropein algycone; HDCM-OA: Hydroxydecarboxymethyl oleuropein aglycone; OA: Oleuropein aglycone; LA: Ligstroside aglycone; OLE: Oleacein; OLC: Oleocanthal.

The loading plot shows the distribution of the different parameters analyzed in our EVOO samples (Figure 2). A location close to the center indicates a lack of difference among the samples. Thus, peroxides, apigenin, and elenolic acid do not differ among samples along the X axis, indicating they were not affected by the RI. In contrast, the EVOO to the right of the X axis (lowest RI) is richer in chlorophylls, carotenes, oleacein, oleocanthal, phenolic alcohols, secoiridoids, and total polyphenols, whereas those on the left (higher RI) are richer in squalene, phenolic acids, and lignans. Regarding the Y axis, the EVOO samples located below (20 °C of malaxation) have a higher content of phenolic acids, tocopherols, secoiridoids, and total phenolics, and those above (25 and 30 °C of malaxation) have higher values of peroxides, acidity, and hydroxytyrosol.

These results agree with the conclusions drawn from the different analyses carried out in this study. Firstly, a lower RI was associated with a higher content of phenolic compounds, chlorophylls, and carotenes. Secondly, regarding the temperature of malaxation, EVOOs produced at 20 °C had a higher content of phenolic compounds and tocopherols, and the peroxide value and free acidity tended to increase with temperature. If we focus on oleocanthal and oleacein (Figure 2), we observe that to obtain an EVOO with high levels of these two compounds, it is important to collect the olives at an early harvest time, so the RI is low.

## 4. Conclusions

The modulation and optimization of the production process, in which malaxation is a key step, can improve the quality and properties of EVOO. In this study, we evaluated how the variation of malaxation temperature and time could affect the quality of EVOO and its content of the most relevant bioactive compounds. The PCA performed revealed that the factor that most influenced our EVOO was the RI, followed by the temperature. Therefore, to assess the effect of the malaxation parameters, the results were adjusted for the RI. According to the results, the EVOO with the highest quality and highest content of phenolic compounds was produced by 30 min. of malaxation at 20 °C. The quality parameters were barely affected by the tested variables, although there was an increasing trend of oxidation with temperature and time. This was in accordance with the variations in the bioactive compounds analyzed. The antioxidant tocopherols and squalene, which protect fatty acids, especially PUFA, from being oxidized, were negatively affected by higher temperatures. Conversely, pigments increased with temperature, resulting in EVOOs with a greener color. Phenolic compounds were the most complex variable analyzed, as their content is influenced by multiple factors, including agronomical, environmental, genetical, and technological factors. Overall, however, when considering all the EVOO samples, longer malaxation at higher temperatures generated oils with a lower phenolic content due to degradation or oxidation. Oleuropein aglycone and ligstroside aglycone decreased, which suggested they were either oxidized or transformed to oleacein and oleocanthal, respectively. Oleacein was not affected by temperature but decreased with time, whereas oleocanthal increased with both parameters. This study shows that the malaxation conditions that most favor some phenolic compounds could not be the best for others. If we aim to obtain an EVOO with high content of oleocanthal and oleacein, 45 min at 25 °C seems to be the most favorable conditions, although the overall results suggested 30 min and 20 °C as the best conditions. Therefore, other factors such as enzymatic activity and the olive RI should also be taken into account to enhance oleocanthal and oleacein levels.

## Figures and Tables

**Table 1:** Quality parameters (K_232_, K_270_, ∆K, peroxide value (PV), and acidity(A)) for all olive oil samples.

Quality Parameter ^1^	Malaxation Treatment ^2^					
	20 °C		25 °C		30 °C	
	30 min	45 min	30 min	45 min	30 min	45 min
K_232_ (≤2.50)	1.49 ± 0.02 ^a^	1.48 ± 0.03 ^a^	1.47 ± 0.06 ^a^	1.61 ± 0.09 ^b^	1.48 ± 0.06 ^a^	1.52 ± 0.02 ^c^
K_270_ (≤0.22)	0.13 ± 0.00 ^a^	0.10 ± 0.00 ^a^	0.08 ± 0.00 ^a^	0.13 ± 0.00 ^a^	0.17 ± 0.01 ^a^	0.16 ± 0.01 ^a^
∆K (≤0.01)	0.0008 ± 0.0007 ^a^	0.0015 ± 0.0002 ^a^	0.0012 ± 0.0003 ^a^	0.0024 ± 0.0003 ^b^	0.0011 ± 0.0008 ^a^	0.0003 ± 0.0002 ^c^
PV (≤20) (mEq O_2_/kg)	3.37 ± 0.20 ^a^	3.64 ± 0.28 ^a^	5.01 ± 0.17 ^a^	4.99 ± 0.20 ^a^	5.13 ± 0.25 ^a^	5.56 ± 0.17 ^a^
A (≤0.8) (g oleic acid/100 g)	0.10 ± 0.01 ^a^	0.11 ± 0.01 ^a^	0.13 ± 0.01 ^b^	0.12 ± 0.01 ^b^	0.12 ± 0.01 ^b^	0.13 ± 0.01 ^b^

^1^ Results are given as “mean ± sd”. For each EVOO sample, there were 3 experimental replicates and 3 analytical replicates. ^2^ Different letters in the same row mean statistically significant differences (*p* < 0.05).

**Table 2:** Concentration of the phenolic compounds identified in the EVOO samples.

Compound ^1^	Concentration (mg/kg Oil) ^2^					
	20 °C		25 °C		30 °C	
	30 min	45 min	30 min	45 min	30 min	45 min
**Flavones**	4.02 ± 0.07	3.89 ± 0.04	3.96 ± 0.07	3.88 ± 0.06	3.69 ± 0.09	4.47 ± 0.06
Apigenin	2.44 ± 0.08	2.36 ± 0.09	2.44 ± 0.06	2.33 ± 0.08	2.10 ± 0.07	2.86 ± 0.16
Luteolin	1.59 ± 0.03	1.52 ± 0.06	1.51 ± 0.02	1.54 ± 0.02	1.58 ± 0.04	1.61 ± 0.06
**Phenolic acids**						
*p*-Coumaric	6.78 ± 0.03	6.86 ± 0.05	6.80 ± 0.04	6.64 ± 0.02	6.66 ± 0.03	6.65 ± 0.01
**Phenolic alcohols**	3.65 ± 0.12	3.75 ± 0.14	3.82 ± 0.08	4.88 ± 0.10	4.96 ± 0.14	4.04 ± 0.03
3,4-DHPEA	0.45 ± 0.02	0.47 ± 0.01	0.60 ± 0.02	0.81 ± 0.06	0.61 ± 0.02	0.66 ± 0.02
3,4-DHPEA-AC I	1.03 ± 0.01	1.01 ± 0.02	1.00 ± 0.02	1.01 ± 0.02	1.00 ± 0.02	0.99 ± 0.02
3,4-DHPEA-AC II	1.18 ± 0.01	1.18 ± 0.03	1.14 ± 0.01	1.20 ± 0.01	1.15 ± 0.02	1.15 ± 0.01
3,4-DHPEA-O-glucoside	0.99 ± 0.09	1.07 ± 0.09	1.10 ± 0.06	1.86 ± 0.10	2.20 ± 0.10	1.27 ± 0.10
**Lignans**						
Pinoresinol	2.04 ± 0.07	2.03 ± 0.09	2.31 ± 0.07	1.86 ± 0.14	1.61 ± 0.01	2.10 ± 0.03
**Secoiridoids**	258.86 ± 5.83	245.58 ± 6.22	261.49 ± 3.78	300.88 ± 4.63	292.83 ± 2.43	281.09 ± 8.66
HDCM-OA	1.29 ± 0.90	1.15 ± 0.01	1.16 ± 0.04	1.19 ± 0.01	1.23 ± 0.07	1.18 ± 0.01
HOA	1.49 ± 0.04	1.49 ± 0.08	1.39 ± 0.05	1.28 ± 0.04	1.26 ± 0.03	1.29 ± 0.02
Lactone	3.75 ± 0.16	3.49 ± 0.13	3.82 ± 0.19	3.01 ± 0.20	3.11 ± 0.09	3.87 ± 0.15
Elenolic acid	10.64 ± 0.22	8.19 ± 0.55	8.18 ± 0.47	8.35 ± 0.46	8.28 ± 0.35	6.21 ± 0.35
Ligstroside aglycone	47.51 ± 1.61	36.27 ± 1.07	30.97 ± 1.68	40.59 ± 1.88	37.48 ± 0.87	25.58 ± 1.49
Oleuropein aglycone	30.68 ± 0.95	25.91 ± 1.00	25.29 ± 1.11	32.02 ± 0.98	31.03 ± 1.21	23.30 ± 0.71
Oleacein	83.38 ± 2.86	76.01 ± 2.68	83.45 ± 3.82	105.12 ± 2.84	105.85 ± 2.41	98.52 ± 3.76
Oleocanthal	80.03 ± 3.78	93.70 ± 3.81	107.59 ± 6.25	109.85 ± 5.13	104.18 ± 2.97	120.98 ± 6.79
**Total phenols**	275.51 ± 5.83	262.10 ± 6.37	278.38 ± 3.72	318.15 ± 4.39	309.74 ± 2.49	298.29 ± 8.57

^1^ 3,4-DHPEA: Hydroxytyrosol; 3,4-DHPEA-AC: Hydroxytyrosol acetate; HDCM-OA: Hydroxydecarboxymethyl oleuropein aglycone; HOA: Hydroxyoleuropein algycone. ^2^ Results are given as “mean ± sd”. For each EVOO sample, there were 3 experimental replicates and 3 analytical replicates.

**Table 3:** Estimated β value and *p*-value for the concentration of total phenols, the phenolic groups, and the major secoiridoids (oleuropein aglycone, oleacein, ligstroside aglycone, and oleocanthal) adjusted for the RI and with a margin of error of 95% over malaxation temperature and time. *p* values and estimated β value for the kernel regression.

Phenolic Group	Comparisons	Estimate β (95% CI) ^1^	*p*-Value
Total phenols	25 vs. 20 °C 30 vs. 20 °C 45 vs. 30 min	−7.95 (−15.08, 0.09) −18.19 (−31.98, −1.58) −8.10 (−15.86, −2.30)	0.036 0.016 0.020
Secoiridoids	25 vs. 20 °C 30 vs. 20 °C 45 vs. 30 min	−8.00 (−15.05, −0.09) −18.25 (−31.87, −1.94) −8.15 (−15.85, −2.37)	0.030 0.020 0.010
Flavones	25 vs. 20 °C 30 vs. 20 °C 45 vs. 30 min	0.08 (−0.04, 0.20) 0.17 (−0.08, 0.41) 0.18 (0.06, 0.35)	0.200 0.170 0.008
Phenolic acids	25 vs. 20 °C 30 vs. 20 °C 45 vs. 30 min	−0.03 (−0.06, 0.003) −0.50 (−0.13, 0.01) 0.03 (0.00, 0.07)	0.120 0.100 0.050
Phenolic alcohols	25 vs. 20 °C 30 vs. 20 °C 45 vs. 30 min	−0.13 (−0.27, −0.01) −0.26 (−0.55, −0.02) −0.23 (−0.38, −0.09)	0.060 0.060 0.002
Lignans	25 vs. 20 °C 30 vs. 20 °C 45 vs. 30 min	0.10 (0.01, 0.20) 0.19 (0.01, 0.40) 0.07 (−0.04, 0.17)	0.040 0.060 0.230
Oleuropein aglycone	25 vs. 20 °C 30 vs. 20 °C 45 vs. 30 min	−4.63 (−5.95, −3.21) −9.35 (−12.06, −6.47) −2.43 (−3.87, −0.97)	<0.001 <0.001 0.001
Oleacein	25 vs. 20 °C 30 vs. 20 °C 45 vs. 30 min	−0.77 (−3.33, 2.1) −2.08 (−6.92, 4.62) −5.75 (−8.02, −3.43)	0.580 0.460 <0.001
Ligstroside aglycone	25 vs. 20 °C 30 vs. 20 °C 45 vs. 30 min	−10.59 (−12.74, −8.2) −21.13 (−25.42, −16.35) −3.58 (−6.93, −0.63)	<0.001 <0.001 0.020
Oleocanthal	25 vs. 20 °C 30 vs. 20 °C 45 vs. 30 min	8.65 (4.82, 12.97) 16.6 (9.2, 25.16) 4.75 (−0.18, 9.45)	<0.001 <0.001 0.070

^1^ Negative β values indicate a decreasing tendency, whereas positive β values indicate an increasing tendency. For each phenolic group or compound *n* = 54 (6 EVOO samples × 3 experimental replicates × 3 analytical replicates).

**Table 4:** Concentration of carotenes, chlorophylls, tocopherols, and squalene in all the olive oil samples produced.

Compound ^1^	Malaxation Treatment					
	20 °C		25 °C		30 °C	
	30 min	45 min	30 min	45 min	30 min	45 min
Carotenes	1.81 ± 0.10	1.69 ± 0.07	1.90 ± 0.05	2.72 ± 0.07	2.58 ± 0.01	2.28 ± 0.12
Chlorophylls	1.58 ± 0.06	1.90 ± 0.10	2.15 ± 0.10	3.28 ± 0.08	3.08 ± 0.27	2.54 ± 0.11
Tocopherols	192.46 ± 9.32	187.23 ± 14.27	166.68 ± 14.08	195.43 ± 11.53	174.39 ± 7.34	179.13 ± 6.64
Squalene	1571.32 ± 42.67	1834.88 ± 69.13	1674.02 ± 91.62	1496.11 ± 108.63	1502.88 ± 124.46	1514.98 ± 53.25

^1^ Results are given as “mean ± sd”. For each EVOO sample, there were 3 experimental replicates and 3 analytical replicates.

## Data Availability

Data is contained within the article.

## Abbreviations

EVOO	Extra-virgin olive oil
RI	Ripening index
PCA	Principal component analysis
PV	Peroxide value
A	Acidity
PUFA	Polyunsaturated fatty acids
PPO	Polyphenol oxidase
POD	Peroxidase
3,4-DHPEA	Hydroxytyrosol
3,4-DHPEA-AC	Hydroxytyrosol acetate
HDCM-OA	Hydroxydecarboxymethyl oleuropein aglycone
HOA	Hydroxyoleuropein aglycone

## Appendix A

**Table A1:** The correspondence between the olive tree and the RI for each EVOO produced.

EVOO Malaxation Treatment			Olive Tree	RI ^1^
Temperature	Time	Replicate		
20 °C	30 min	1	P4-G10	1.44
		2	P4-G10	1.44
		3	P4-G9	2.20
	45 min	1	P4-G9	2.20
		2	P4-G11/12	1.54
		3	P4-G11/12	1.54
25 °C	30 min	1	P4-G11/12	1.54
		2	P4-G11/12	1.54
		3	P4-G11/12	1.54
	45 min	1	P4-G4/6	1.20
		2	P4-G4/6	1.20
		3	P4-G4/6	1.20
30 °C	30 min	1	P4-G4/6	1.20
		2	P4-G4/6	1.20
		3	P4-G1/2/3	2.26
	45 min	1	P4-G15/16	1.16
		2	P4-G15/16	1.16
		3	P4-G15/16	1.16

^1^ The RI was calculated with a representative olive sample of each different olive tree. Therefore, EVOOs produced with olives belonging to different olive trees may have different RIs.
